# Functionalized Gels for Environmental Applications

**DOI:** 10.3390/gels9100818

**Published:** 2023-10-14

**Authors:** Luca Burratti, Paolo Prosposito, Iole Venditti

**Affiliations:** 1 Sciences Department, Roma Tre University, Via della Vasca Navale 79, 00146 Rome, Italy; luca.burratti@uniroma3.it (L.B.); iole.venditti@uniroma3.it (I.V.); 2 Department of Industrial Engineering, University of Rome Tor Vergata, Via del Politecnico 1, 00133 Rome, Italy

A gel is a type of material that exhibits a semi-solid, jelly-like state, characterized by a three-dimensional network of interconnected particles or molecules dispersed within a liquid or solid medium. This network structure provides the gel with unique physical properties, including the ability to maintain its shape while possessing a high degree of flexibility and deformability. They are distinguished by their capacity to hold a significant amount of liquid within their structure, making them valuable in applications ranging from personal care products (such as hair gel and skincare formulations) to biomedical materials (like hydrogels for tissue engineering) and technological applications (such as aerogels for insulation and catalyst supports). Gel-based systems have demonstrated their utility in diverse environmental contexts, such as the treatment of contaminated water bodies, the decontamination of soil, and the purification of air. The wide-ranging versatility of gel in environmental applications is exemplified by their efficacy in removing a diverse array of contaminants, such as heavy metals, organic pollutants, dyes, and even emerging contaminants like pharmaceuticals and microplastics. The selective adsorption of these pollutants arises from a combination of chemical interactions, such as ion exchange, coordination, and electrostatic attraction, as well as physical mechanisms like diffusion and adsorption.

This Special Issue, entitled “Functionalized Gels for Environmental Applications”, in the *Gels* journal includes nine original articles and one review. These manuscripts address different aspects of environmental monitoring/remediation and they provide an interesting overview of the fields covered by research on gels, offering valuable insights into the challenges and prospects that lie ahead for future advancements in the field.

The adsorption capacity of hydrogels toward contaminants can be enhanced by modifying them via various techniques; for example, in the work conducted by M. Klučáková, an agarose hydrogel is enriched by chitosan as an active compound for the adsorption of dyes [1]. The author reports that the adsorption capacity of modified agarose is several times higher in comparison with pure agarose hydrogel and that this behavior is also found at different pH values (3, 7 and 11), certifying the success of the modification. The author asserts that the increase in the dyes’ adsorption is due to the electrostatic interactions between the amino group of chitosan and the sulfonic group of dyes, which leads to the formation of distinct dye layers on the surface of the modified hydrogel. From the perspective of rheology, the addition of chitosan results in changes in storage and loss moduli, which can be exhibited on a “more liquid” character of enriched hydrogels. This can contribute to an increase in the effective diffusion coefficients for hydrogels with a higher content of chitosan [2].

Another example of how a properly designed hydrogel is able to remove methylene blue and diclofenac from water is represented by the research of A. Fortunato and M. Mba [3]. In this work, a tetrapeptide–pyrene conjugate is designed to form hydrogels under controlled acidic conditions. The main results indicate that the methylene blue adsorption is guided by the availability of adsorption sites, while in the case of diclofenac, the concentration is the driving force of the process. In the case of methylene blue, the nature of the dye–hydrogel interactions are explained: first, the dye is adsorbed as a monomer (the authors hypothesize an electrostatic interaction) and successively, at increasing concentrations as the electrostatic adsorption sites are depleted, dimerization on the hydrogel surface occurs. The removal efficiencies (that depend on the initial concentration of the pollutants) for methylene blue and diclofenac are in the range of 90–100% and 53–89%, respectively. 

The presence of diclofenac as a water contaminant poses a risk to human health; therefore, it is vital that it can be easily removed from polluted water. The research carried out by M. Chelu et al. [4] constitutes a valid in-depth study on the removal of this pharmaceutical product. In this case, the hydrogel is based on a mixture of chitosan, polyethylene glycol and xanthan gum (CPX), and it is prepared via a green method. The authors characterize the materials well and find that the swelling properties remain unchanged in a wide range of pH (3–9). Moreover, the adsorption capacity study reveals that the adsorbent hydrogel reaches the adsorption capacity (172.41 mg/g) at the highest adsorbent amount (200 mg) after 350 min. Finally, the data obtained in the kinetic study reveal that the used adsorbent may possess great applicative potential in environmental applications as a water cleaning agent.

Heavy metal ions are another class of water pollutants, and their presence in drinkable water poses serious problems to the health of living beings (plants and animals); therefore, it is essential that water can be purified in a simple and effective way. Burratti et al. [5] successfully develop a hydrogel-based filter for removing Pb(II) ions from water. Poly(ethylene glycol) diacrylate (PEGDA) hydrogels, modified with luminescent silver nanoclusters (AgNCs), are synthesized through a photo-crosslinking procedure. This type of filter is able to remove between 80% to 90% of the lead impurity. Their experimental findings suggest that the adsorption of Pb(II) onto the modified filter is predominantly driven by favorable chemisorption. Considering its exceptional contaminant uptake capacity and cost-effectiveness, this hybrid system exhibits significant potential as an adsorbent material for the efficient removal of Pb(II) ions from aqueous environments.

The versatility and unique qualities of thermo-responsive polymeric systems have led to the application of these materials in a multitude of fields. Environmental remediation can significantly benefit from the utilization of innovative and smart materials. Notably, the multifaceted nature of poly(N-isopropylacrylamide) (PNIPAAm) systems, incorporating PNIPAAm copolymerized with diverse cationic comonomers, holds the potential to selectively target and attract negatively charged contaminants, such as perfluorooctanoic acid (PFOA). In the study presented by E. M. Frazar et al. [6], the synthesis of a variety of thermo-responsive cationic hydrogels is carried out. In this work, the effect of pH on the hydrogel swelling behavior is studied and found to be insignificant for PNIPAAm and hydrogels containing loading percentages of 1 and 5 mol% cationic comonomer. The inclusion of cationic comonomers, however, alters the hydrogel swelling capacity, mostly due to losses in the thermo-responsive behavior as the comonomer amount is increased. The adsorption of PFOA is inversely related to buffered aqueous pH, while the cationic monomer type has little noticeable consequence in the buffered solutions. These insights gained from hydrogel performance under variable pH, buffer, temperature, and comonomer compositions provide us with a deeper understanding of which polymer functionalities are most beneficial when designing materials for the remediation of perfluoroalkyl substances in aqueous environments.

Air contaminants, also known as air pollutants, are substances or particles present in the air that can have harmful effects on human health, the environment, or both. Efforts to reduce air contaminants often involve regulatory measures, technological advancements, and changes in behavior to minimize emissions and exposure. A very interesting study is presented by P. Chesler et al. [7], who develop two sensors, one based on cobalt and one based on copper, to detect low concentrations of methane. The authors synthesize sensitive films on an alumina substrate, with gold or platinum interdigital electrodes (IDE) printed onto the alumina surface, using the sol–gel technique. The fabricated sensors exhibit stability, partial selectivity, and the ability to detect low concentrations of methane (5 ppm) with a rapid response time of 250 s and complete recovery within the same timeframe. Some response to interfering species (CO_2_ and humidity) is observed, but it is relatively modest, counting for approximately 50% of the sensor’s response to methane. The cobalt-based sensor demonstrates superior selectivity, particularly at elevated methane concentrations.

Nowadays, the detection and removal of the CO_2_ that naturally occurs in the Earth’s atmosphere and is produced abundantly by many industrial processes in high demand, since excessive concentrations can pose hazards to both human health and the environment. Within this framework, H. Choi et al. [8] successfully fabricate novel macroporous hydrogel particles comprising hyperbranched poly(amidoamine)s (HPAMAM) utilizing the oil-in-water-in-oil (O/W/O) suspension polymerization technique. This method, known for conferring a porous architecture to microparticles, results in hydrogel particles with a rich abundance of amine groups embedded within the polymer matrix. Therefore, these synthesized hydrogel particles demonstrate remarkable CO_2_ absorption capabilities, with an absorption capacity of 104 mg/g, and exhibit rapid absorption rates in rigorous packed-column tests.

Until now, the topics addressed have concerned the removal of pollutants of various kinds, but sometimes the material employed for purification can be fouled by organic/inorganic substances, consequently reducing the performance of the material enormously. Developing solutions to avoid this type of problem is desirable. An interesting study carried out by S. Sfameni et al. [9] concerns the development of hybrid functional coatings for anti-biofouling and foul-release activity. Here, silica-based materials are prepared using two alkoxysilane cross-linkers containing epoxy and amine groups in combination with two functional fluoro-silanes, featuring well-known hydro repellent and anti-corrosion properties. The efficacy of fouling the release properties is assessed by subjecting the material to various microbial suspensions in seawater-based solutions and within natural seawater microcosms. The newly formulated fluorinated coatings exhibit antimicrobial capabilities. Notably, no biocidal effects are observed on the microorganisms, specifically bacteria.

Finally, in their review, Z. Darban et al. [10] describe the methods employed in order to recycle wastewater by exploiting hydrogel-based adsorbent materials. The synthesis techniques and adsorption mechanisms are also explored, focusing on the regeneration, recovery, and reuse of modified hydrogels.

In conclusion, because the evolution of technology and materials in this area is rapid and extensive, as Guest Editors we realize that it is limiting to present them in a single volume. However, the multidisciplinary nature and high quality of the articles allow us to provide readers with an updated and broad panorama regarding functionalized gels for environmental applications, which we are certain will arouse great interest.
